# Supplementary material for: Inferring gene-regulatory networks using epigenomic priors
Source: iScience. 2026 Feb 28;29(4):115165. doi: 10.1016/j.isci.2026.115165 (PMC13053756; doi:10.1016/j.isci.2026.115165)
Supplement: Document S1. Figures S1–S8 and Tables S1–S10 [file mmc1.pdf]

iScience, Volume *29*

Supplemental information

Inferring gene-regulatory networks using epigenomic priors

Thomas E. Bartlett, Melodie Li, Chenyu Song, Yuche Gao, and Qiulin Huang

Supplementary figures and tables

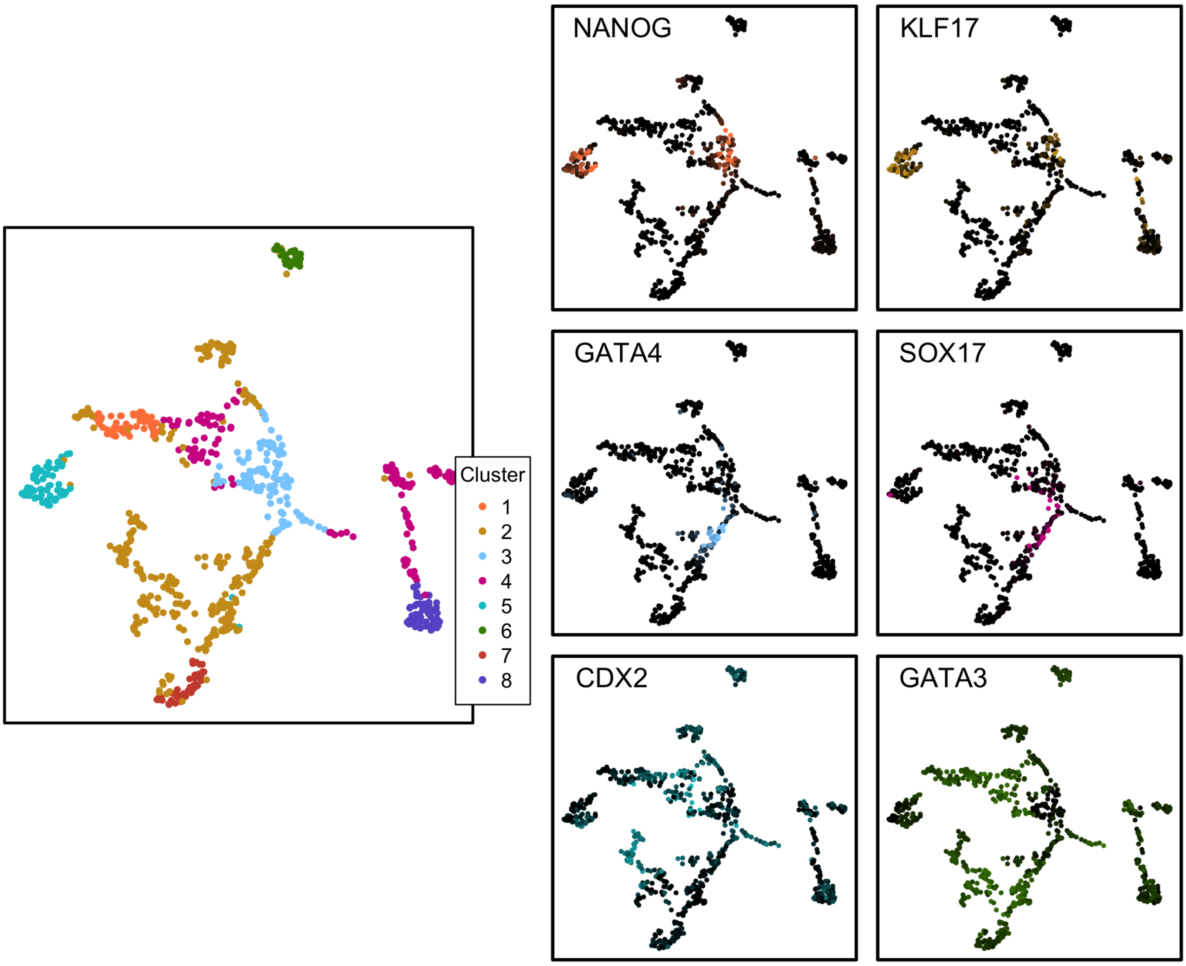

Figure S1: *Related to Figures 4 and 5. UMAP projections from the Laplacian eigenspace (UMAP-LE) [1, 2] show GMM-LE clusters and expression levels of validating marker genes for all cells from 5, 6, and 7 days post-fertilisation for the human embryonic development dataset [3].*

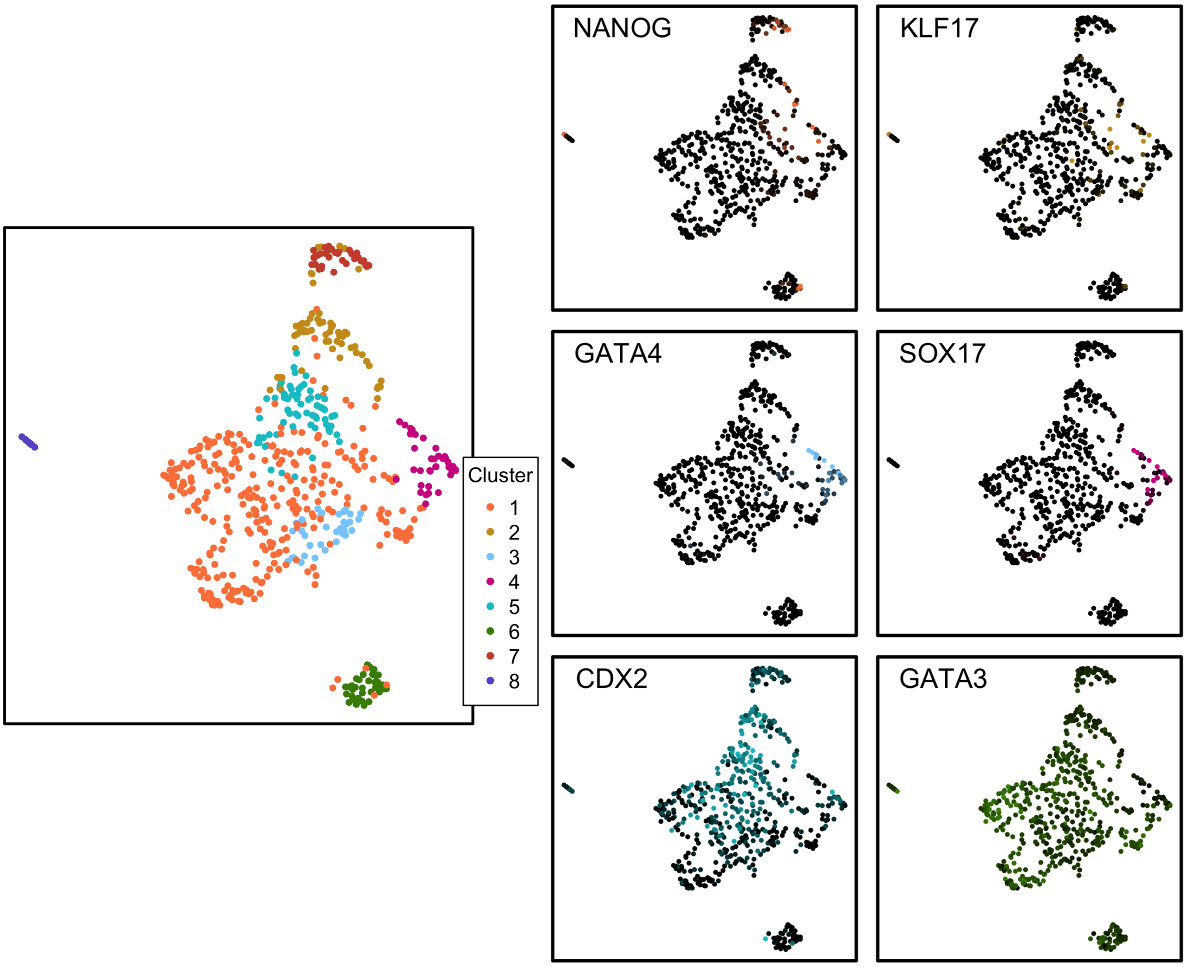

Figure S2: *Related to Figures 4 and 5. UMAP projections from the Laplacian eigenspace (UMAP-LE) [1, 2] show GMM-LE clusters and expression levels of validating marker genes, applied to cluster 2 from Figure S1, for the human embryonic development dataset [3].*

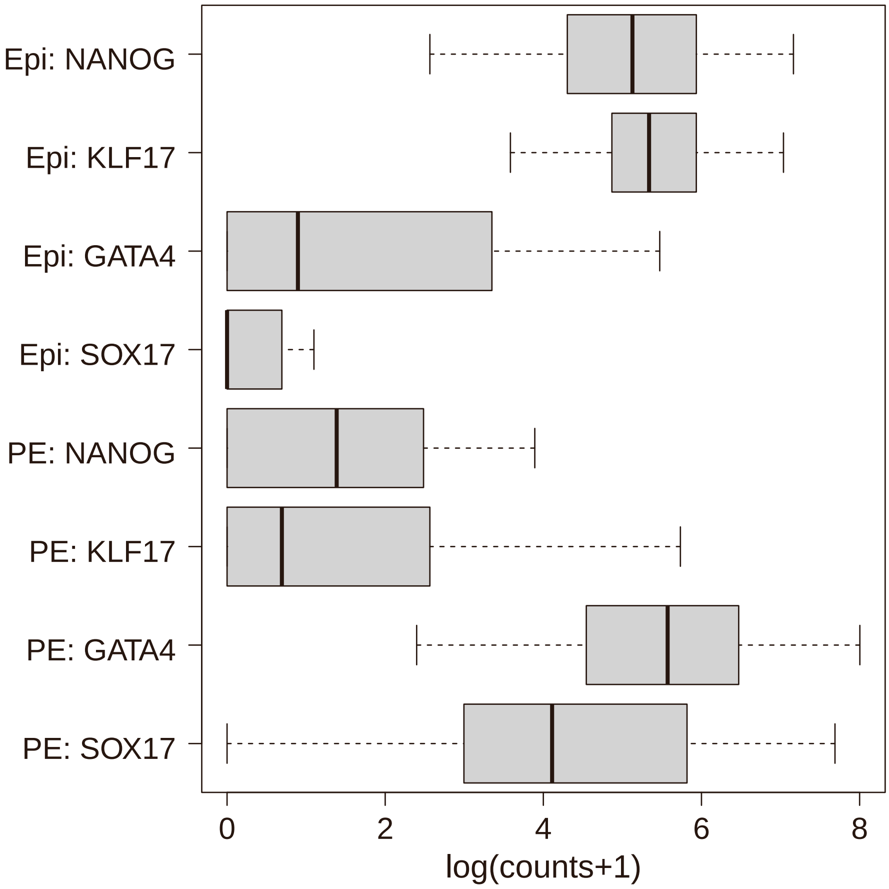

Figure S3: *Related to Figures 4 and 5. Boxplots showing marker genes for Epi (epiblast), validating the 68 Epi cells identified as cluster 5 in Figure S1, and for the PrE (primitive endoderm), validating the 37 PrE cells identified as cluster 4 in Figure S2, for the human embryonic development dataset [3].*

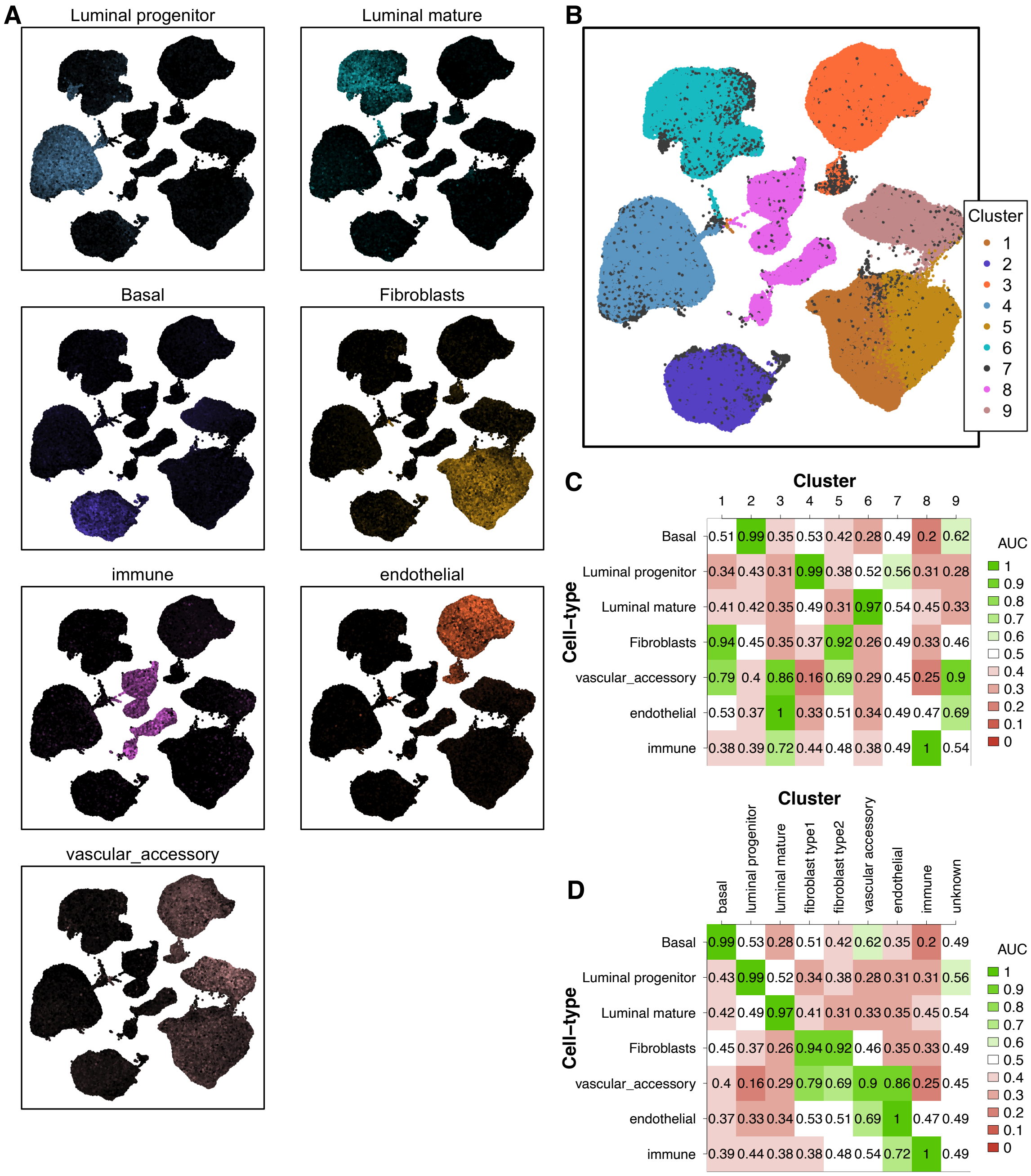

Figure S4: *Related to Figure 7. Unsupervised learning and classification of unknown cell-types in the combined breast-cancer at-risk datasets [4–10]. UMAP projections from the Laplacian eigenspace (UMAP-LE) [1,2] show (a) mean expression levels of validating marker genes and (b) GMM-LE clusters for all cells. In (c) the AUC statistic is calculated (over cells) to compare membership of each cluster, with mean expression level of marker-genes for each cell-type. I.e., the AUC statistic quantifies how well the mean marker-gene expression for a cell-type predicts membership of a cluster. This allows automatic cell-type identification via maximum-AUC criterion as shown in (d), as well as assessment of clustering accuracy. These cell-type definitions are used subsequently in all analyses based on the combined breast cancer at-risk datasets.*

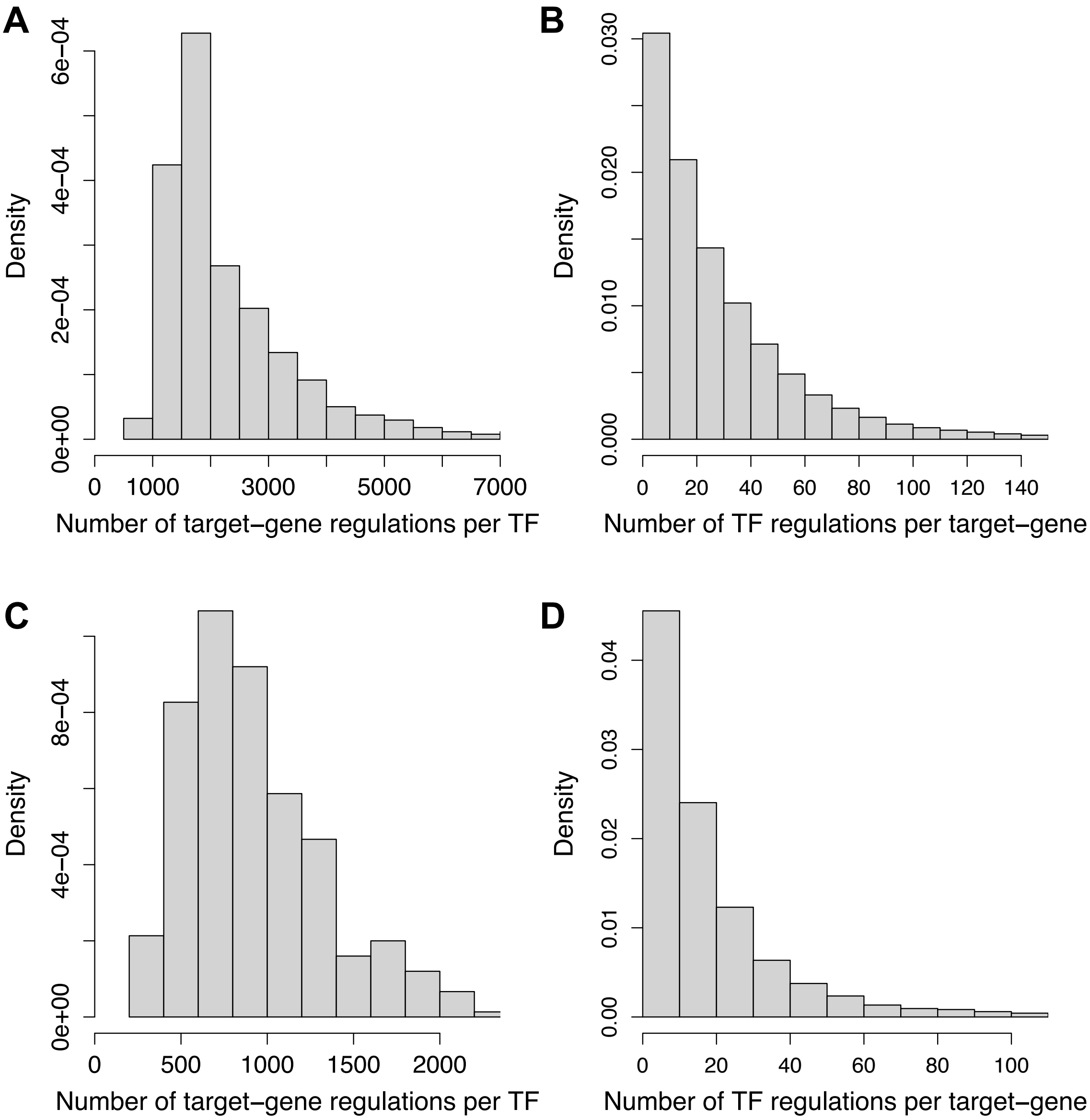

Figure S5: *Related to Figures 5 and 7. Histograms showing the distributions of the numbers of regulations for TFs, and of regulators for target genes. The histograms in (a) and (c) show the numbers of target-gene regulations for each TF, and in (b) and (d) the number of TF regulators for each target-gene, in the epigenomic prior networks for (a) and (b) the breast cancer at-risk dataset, and (c) and (d) the human embryonic development dataset.*

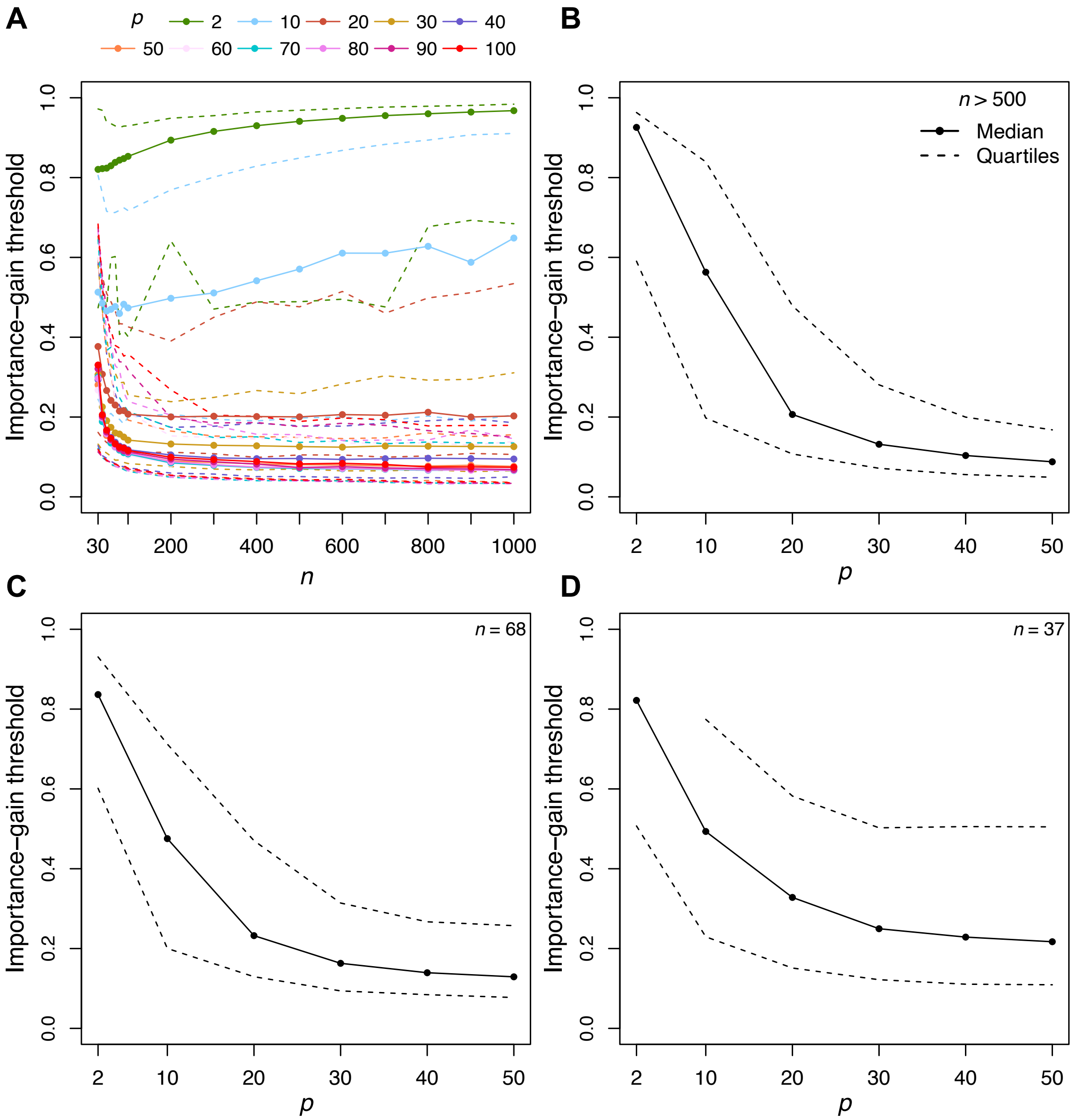

Figure S6: *Related to Figures 5 and 7. Simulation study results, to inform setting the xgboost importance-gain threshold. (a) Synthetic data with known ground-truth network structure are used to select the optimal importance-gain threshold (assessed by maximum-AUC criterion) for each target-gene in 250 synthetic datasets, for varying* p *and* n*; solid lines show means, and dashed lines show lower and upper quartiles. For* $p > 50$ *there is little change in the optimal threshold, so we use the same threshold for all* $p > 50$*. Similarly for* $n > 500$ *(which includes all GRN inference based on the combined breast-cancer at-risk dataset), there is little change in the optimal threshold, so we use the same threshold for all* $n > 500$*, as shown in (b). For* $n < 500$*, we use thresholds specific to the sample size:* $n = 68$ *for epiblast, and* $n = 37$ *for primitive endoderm cells, as shown in (c) and (d) respectively.*

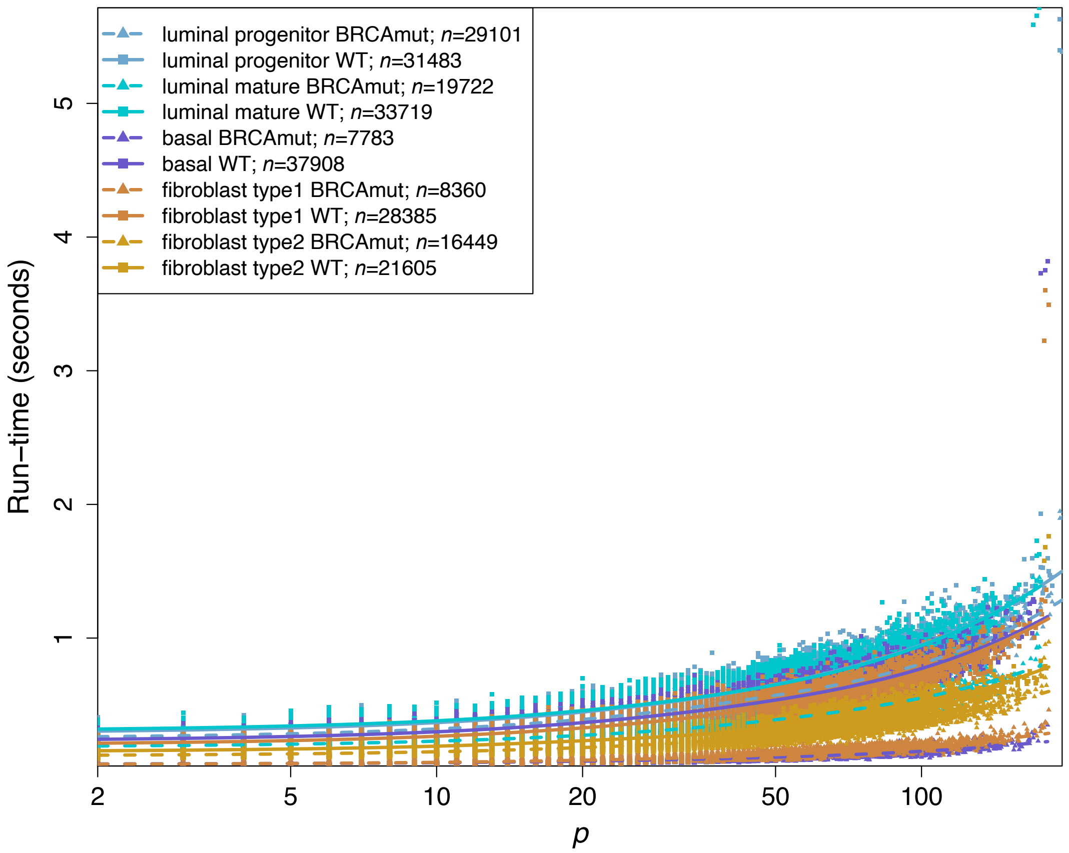

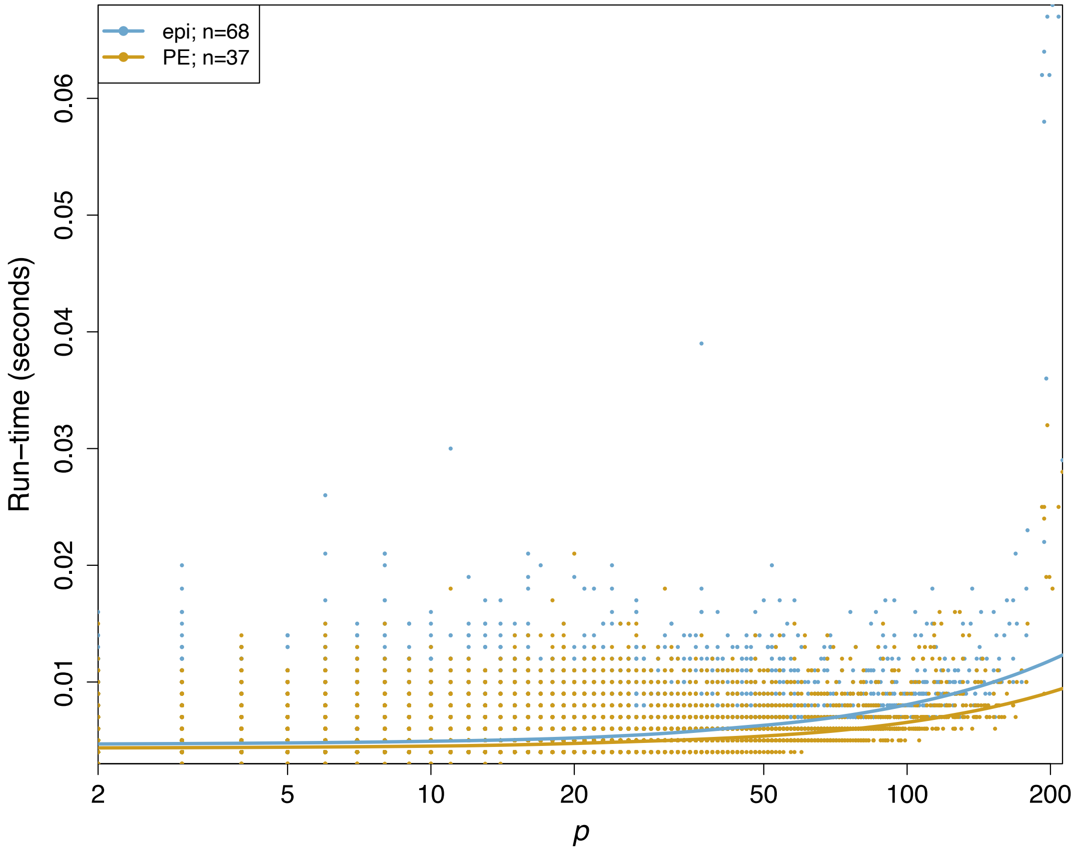

Figure S7: *Related to Figures 5 and 7. Runtime is plotted against number of TF predictors* p *in each target-gene random-forests model, for each GRN inference carried out for the combined breast-cancer at-risk dataset, and the human embryonic development dataset. Each point plotted corresponds to one random-forests model fit for one target-gene. Trend-lines are fitted with lowess regression.*

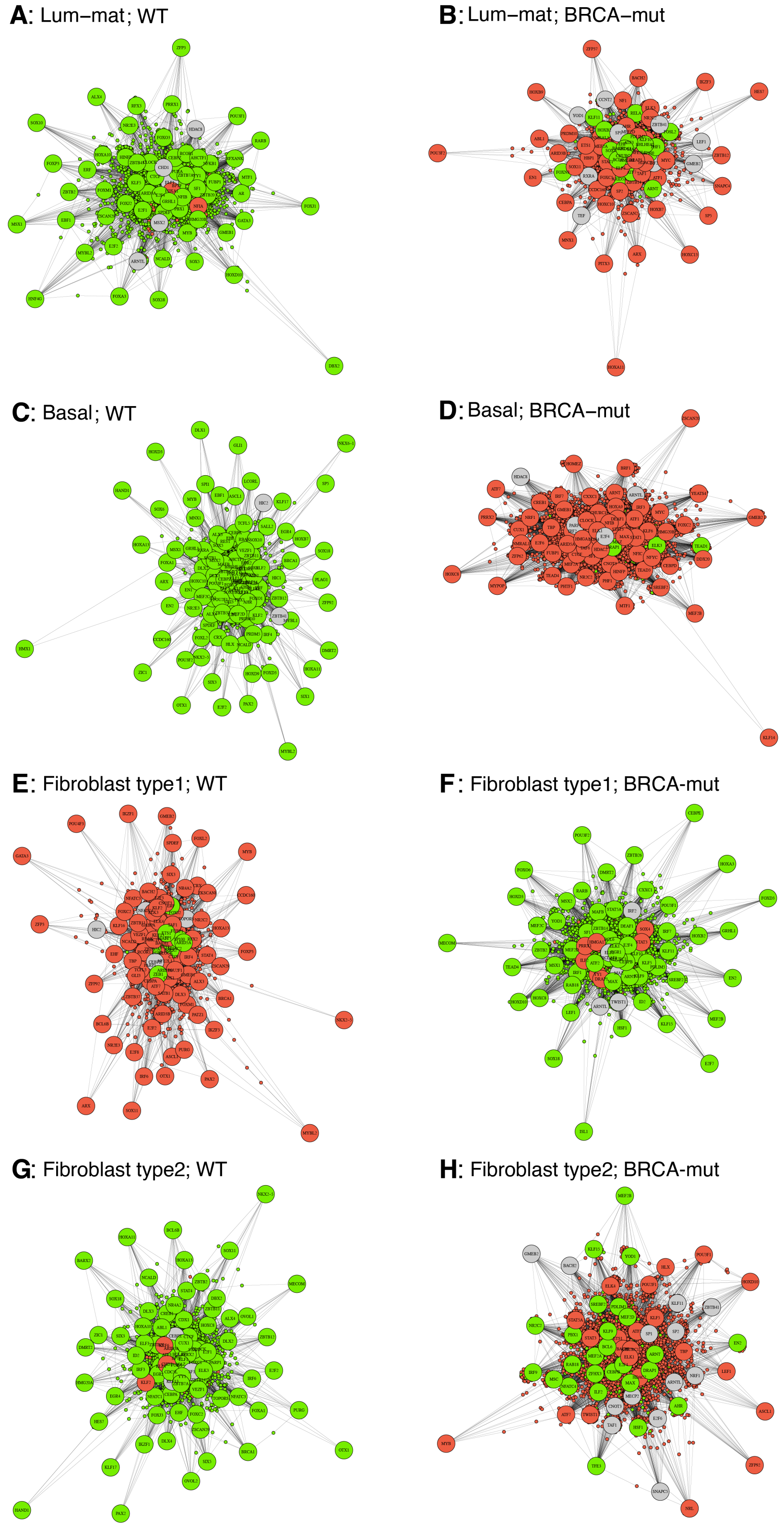

Figure S8: *Related to Figure 7. Differential regulation networks inferred for the the breast-cancer at-risk data. Shown in (a) and (b) mature luminal cells (lum-mat), in (c) and (d) basal cells, and in (e), (f), (g), and (h) fibroblasts, for (a), (c), (e), and (g) BRCA1/2 wild-type (WT), and (b), (d), (f), and (h) BRCA1/2 mutation carriers (BRCAmut). Large circles indicate significantly differentially-regulating (diff-reg) transcription factors (Table S3, Table S4, Table S5, Table S6, Table S7); green and red indicate (respectively) significantly upregulated and downregulated TFs and genes; significance level is FDR* $p\text{-}val < 0.05$, *(t-test, Benjamini-Hochberg adjustment).*

TF	OR	*p*-val	*p*-val RND
E4F1	1110 (851 - 1460)	<0.001	<0.001
BHLHE40	858 (644 - 1190)	<0.001	<0.001
CEBPZ	844 (715 - 928)	<0.001	<0.001
NFYA	738 (669 - 825)	<0.001	<0.001
NFYC	733 (629 - 825)	<0.001	<0.001
ATF1	562 (456 - 716)	<0.001	<0.001
MITF	482 (394 - 573)	<0.001	<0.001
ISX	459 (316 - 629)	<0.001	<0.001
POU5F1	409 (311 - 530)	<0.001	<0.001
RFX2	394 (333 - 472)	<0.001	<0.001
CREM	386 (309 - 475)	<0.001	<0.001
EN2	347 (231 - 505)	<0.001	<0.001
FOXI1	331 (286 - 372)	<0.001	<0.001
BARHL1	330 (172 - 588)	<0.001	<0.001
LHX6	296 (239 - 372)	<0.001	<0.001
TFE3	286 (237 - 338)	<0.001	<0.001
MAX	257 (215 - 305)	<0.001	<0.001
ATF7	257 (199 - 330)	<0.001	<0.001
SOX9	256 (225 - 286)	<0.001	<0.001
KLF14	246 (230 - 262)	<0.001	<0.001
HMBOX1	243 (149 - 376)	<0.001	<0.001
ELK1	240 (214 - 274)	<0.001	<0.001
KLF11	233 (217 - 252)	<0.001	<0.001
GMEB2	230 (158 - 331)	<0.001	<0.001
CREB1	229 (191 - 272)	<0.001	<0.001
SP2	218 (207 - 233)	<0.001	<0.001
NRF1	212 (193 - 232)	<0.001	<0.001
E2F4	203 (191 - 218)	<0.001	<0.001
GSC	200 (140 - 282)	<0.001	<0.001
ZBTB14	190 (172 - 211)	<0.001	<0.001
ATF2	184 (157 - 217)	<0.001	<0.001
ATF3	183 (153 - 215)	<0.001	<0.001
OVOL1	173 (98.6 - 287)	<0.001	<0.001
EGR1	173 (164 - 184)	<0.001	<0.001
YY1	166 (140 - 199)	<0.001	<0.001
PAX4	162 (101 - 250)	<0.001	<0.001
SNAI1	154 (118 - 201)	<0.001	<0.001
ERG	154 (137 - 174)	<0.001	<0.001
ISL2	154 (108 - 217)	<0.001	<0.001
BARX1	147 (107 - 198)	<0.001	<0.001
E2F2	143 (121 - 167)	<0.001	<0.001
HOXA9	141 (62.4 - 286)	<0.001	<0.001
EMX1	139 (89.3 - 209)	<0.001	<0.001
ZBTB33	135 (104 - 175)	<0.001	<0.001
EGR4	127 (114 - 140)	<0.001	<0.001
E2F1	124 (114 - 135)	<0.001	<0.001
ETV6	123 (109 - 139)	<0.001	<0.001
ELF1	116 (105 - 128)	<0.001	<0.001
HBP1	115 (70.5 - 178)	<0.001	<0.001
SPDEF	112 (90.6 - 137)	<0.001	<0.001
SOX13	111 (65.1 - 180)	<0.001	<0.001
JUN	111 (89.8 - 137)	<0.001	<0.001
ELK3	111 (91.1 - 134)	<0.001	<0.001
HOXD13	110 (77.9 - 153)	<0.001	<0.001
EHF	110 (97.6 - 124)	<0.001	<0.001
AHR	109 (65.9 - 172)	<0.001	<0.001
ERF	107 (76.5 - 147)	<0.001	<0.001
SREBF2	102 (75.7 - 135)	<0.001	<0.001
HOXA13	102 (70.2 - 144)	<0.001	<0.001
PRRX2	100 (72.1 - 137)	<0.001	<0.001
BSX	97 (69 - 134)	<0.001	<0.001
EVX1	93.9 (46.1 - 172)	<0.001	<0.001
TAF1	91.2 (83.3 - 99.8)	<0.001	<0.001
CDX1	88.5 (61.7 - 124)	<0.001	<0.001
FOXN3	87.2 (51.6 - 139)	<0.001	<0.001
LHX2	86.8 (65.6 - 113)	<0.001	<0.001
KLF16	83.3 (73.7 - 93.4)	<0.001	<0.001
PRRX1	83.2 (65.1 - 105)	<0.001	<0.001
ETS1	83 (69.4 - 98.5)	<0.001	<0.001
CDC5L	82.2 (34.5 - 169)	<0.001	<0.001
MEF2D	81.9 (52.5 - 122)	<0.001	<0.001
ARNT	80.2 (51.4 - 120)	<0.001	<0.001
PBX1	79.7 (61.3 - 102)	<0.001	<0.001
NKX2-3	78 (43.9 - 129)	<0.001	<0.001
WT1	77.6 (70.5 - 85.6)	<0.001	<0.001
NKX6-2	77.3 (48 - 119)	<0.001	<0.001
LHX8	77 (46.3 - 122)	<0.001	<0.001
LHX3	75 (39.1 - 132)	<0.001	<0.001
BARX2	74 (45.7 - 114)	<0.001	<0.001
TBP	72.6 (46.5 - 109)	<0.001	<0.001
TEF	71.3 (40.9 - 116)	<0.001	<0.001
IRF2	67.1 (52.6 - 85)	<0.001	<0.001
SOX5	66.9 (44.6 - 97.2)	<0.001	<0.001
MEF2C	66.5 (41.1 - 103)	<0.001	<0.001
CEBPB	65.9 (42.1 - 99.1)	<0.001	<0.001
FOXO1	63.7 (39.5 - 97.9)	<0.001	<0.001
GATA1	63 (46.4 - 83.9)	<0.001	<0.001
ARX	62.5 (36.2 - 102)	<0.001	<0.001
CUX1	62 (36.4 - 100)	<0.001	<0.001
ZSCAN29	61.6 (34.3 - 103)	<0.001	<0.001
MSX2	60.9 (39.1 - 91.1)	<0.001	<0.001
ZFHX3	57.9 (26.1 - 112)	<0.001	<0.001
LMX1A	57.7 (33.2 - 93.8)	<0.001	<0.001
POU4F3	56.4 (29.5 - 99.1)	<0.001	<0.001
FOXJ3	55 (41.1 - 72.4)	<0.001	<0.001
CEBPA	52.8 (36.1 - 75)	<0.001	<0.001
CDX2	52.5 (38.9 - 69.6)	<0.001	<0.001
IRF7	52.4 (38.5 - 69.7)	<0.001	<0.001
ALX3	52 (38.3 - 69.6)	<0.001	<0.001
KLF8	51.8 (39.6 - 66.9)	<0.001	<0.001
STAT1	51.2 (41.6 - 62.6)	<0.001	<0.001
CLOCK	50.7 (36.5 - 69.1)	<0.001	<0.001
HOXA10	50.6 (31 - 78.8)	<0.001	<0.001
ONECUT2	49.7 (21 - 101)	<0.001	<0.001
FOXG1	48.8 (36 - 64.9)	<0.001	<0.001
ZBTB26	48.2 (34.2 - 66.4)	<0.001	<0.001
SREBF1	48.2 (29.1 - 75.1)	<0.001	<0.001
FOSL2	47.8 (29.9 - 72.4)	<0.001	<0.001
POU2F1	47 (34.9 - 62.3)	<0.001	<0.001
ELF3	46.3 (39.9 - 53.8)	<0.001	<0.001
MZF1	46.1 (38.7 - 54.9)	<0.001	<0.001
RXRA	45.5 (38.1 - 54.1)	<0.001	<0.001
HOXD10	45.2 (16.2 - 102)	<0.001	<0.001
HOXC10	44.8 (16.1 - 101)	<0.001	<0.001
DLX3	44.1 (27.1 - 68.6)	<0.001	<0.001
OTX1	43.8 (27.7 - 66)	<0.001	<0.001
OLIG3	42.8 (18.2 - 86.3)	<0.001	<0.001
DLX1	42.2 (13.6 - 99.9)	<0.001	<0.001
CRX	41.7 (27.3 - 61.5)	<0.001	<0.001
SOX14	41.7 (16.6 - 87.4)	<0.001	<0.001
REST	41.6 (23.5 - 68.5)	<0.001	<0.001
BPTF	38.9 (26.9 - 54.3)	<0.001	<0.001
GLI2	37.6 (28 - 49.5)	<0.001	<0.001
HOXD3	37.3 (20.1 - 63.7)	<0.001	<0.001
NR1H4	37 (27.7 - 48.5)	<0.001	<0.001
VSX2	36.9 (20.8 - 60.8)	<0.001	<0.001
EN1	36.6 (20.1 - 61.6)	<0.001	<0.001
GBX2	36.5 (20.6 - 60.3)	<0.001	<0.001
FOXA1	36.5 (27.3 - 48)	<0.001	<0.001
BRCA1	34.6 (23.5 - 49.3)	<0.001	<0.001
BCL6	34.3 (23 - 49.3)	<0.001	<0.001
ALX1	33.8 (15.2 - 65.5)	<0.001	<0.001
FOXD2	33.5 (19 - 55.1)	<0.001	<0.001
ONECUT3	33.5 (4.01 - 123)	0.00176	<0.001
PAX3	33.1 (14.1 - 67)	<0.001	<0.001
ZBTB12	33 (18.9 - 53.8)	<0.001	<0.001
RARG	32.5 (25.3 - 41.3)	<0.001	<0.001
SOX10	31.5 (25.3 - 38.7)	<0.001	<0.001
LHX1	29.3 (13.8 - 55)	<0.001	<0.001
FOXC2	26.8 (17.8 - 38.8)	<0.001	<0.001
FOXM1	26.6 (15.6 - 42.4)	<0.001	<0.001
LEF1	26.3 (19.2 - 35.2)	<0.001	<0.001
POU3F2	25.8 (16.8 - 37.9)	<0.001	<0.001
FOXC1	25.5 (18.1 - 34.8)	<0.001	<0.001
SIX1	25.4 (9.25 - 55.9)	<0.001	<0.001
TLX2	24.8 (6.7 - 64.6)	<0.001	<0.001
POU3F1	24.7 (16.3 - 36.2)	<0.001	<0.001
SOX7	24.5 (2.94 - 90.6)	0.00321	<0.001
DLX2	24.2 (10.2 - 49)	<0.001	<0.001
HOXB7	23.9 (6.44 - 62.4)	<0.001	<0.001
SCRT1	23.6 (13.4 - 38.7)	<0.001	<0.001
IRF4	23.1 (11.5 - 41.8)	<0.001	<0.001
RARB	22.5 (14.6 - 33.4)	<0.001	<0.001
ALX4	22.2 (8.04 - 49.2)	<0.001	<0.001
FOXL1	22 (12.7 - 35.6)	<0.001	<0.001
PROP1	21.4 (8.53 - 44.7)	<0.001	<0.001
NR2E1	20.6 (1 - 117)	0.0476	0.012
STAT3	19.6 (4.01 - 57.9)	<0.001	<0.001
SOX1	14.9 (4.79 - 35.5)	<0.001	<0.001
NR2E3	13.5 (6.14 - 25.9)	<0.001	<0.001
FOXJ2	13.4 (6.39 - 24.8)	<0.001	<0.001
HESX1	13.4 (3.62 - 34.6)	<0.001	<0.001
NFE2L1	13.3 (7 - 23)	<0.001	<0.001
HMGA1	13.2 (4.25 - 31.5)	<0.001	<0.001
GRHL1	12 (5.44 - 22.9)	<0.001	<0.001
PHOX2B	11.9 (1.43 - 43.7)	0.0128	<0.001
DLX4	11 (2.25 - 32.8)	0.00282	<0.001
NANOG	9.57 (3.83 - 19.8)	<0.001	<0.001
MYBL2	1 (1 - 135)	1	1
HOXB9	1 (1 - 48.9)	1	1

Table S1: *Related to Figure 3. Odds-ratios (with 95% C.I.s) resulting from comparison of the overlap between the DNA regions inferred as regulated by 170 TFs in the inner cell mass (ICM) according to both the modified-lever-cistarget (MLC) method and the RGT-HINT [11] method, together with significance p-vals calculated according to Fisher's exact test, as well as p-vals calculated by randomising the chromosome data and comparing the resulting resampled odd-ratios with the observed odds-ratios.*

A

TF	mean diff-reg stat	p-val	adj p-val
E2F1	0.00756	<0.001	<0.001
BCLAF1	0.0045	<0.001	<0.001
NANOG	0.0043	<0.001	<0.001
ETS1	0.00292	<0.001	<0.001
ARX	0.00265	<0.001	<0.001
IRF4	0.00245	<0.001	<0.001
KLF17	0.00198	<0.001	<0.001
BRCA1	0.00172	<0.001	<0.001
KLF11	0.00162	<0.001	<0.001
LHX8	0.00159	<0.001	<0.001
KLF4	0.00159	<0.001	<0.001
DLX3	0.00159	0.001	0.005
MZF1	0.00159	<0.001	<0.001
ARID5A	0.00152	<0.001	<0.001
FOXC1	0.00145	<0.001	0.003
GLI2	0.00129	<0.001	<0.001
ALX1	0.00124	0.017	0.047
BBX	0.00123	<0.001	0.002
CNOT3	0.00114	<0.001	0.002
KLF2	0.00112	<0.001	<0.001
HMGA1	0.0011	0.011	0.034
SF1	0.00108	<0.001	0.003
PBX1	0.00107	<0.001	<0.001
GRHL1	0.00107	<0.001	0.002
HDAC2	0.00104	0.008	0.027
NRF1	0.000995	0.004	0.014
CXXC1	0.000949	0.002	0.007
MEF2D	0.000906	<0.001	<0.001
POU3F1	0.000869	<0.001	<0.001
DLX4	0.000848	<0.001	0.002
CDX1	0.000838	0.012	0.035
SOX1	0.000795	<0.001	<0.001
GATA2	0.000776	0.004	0.014
BRF1	0.000761	0.014	0.04
SOX15	0.000759	<0.001	<0.001
GBX2	0.000759	<0.001	0.002
PATZ1	0.000741	0.001	0.006
YBX1	0.000736	0.012	0.035
FOXN3	0.000734	<0.001	0.002
FOXG1	0.000713	<0.001	<0.001
KLF1	0.000711	<0.001	<0.001
ELF3	0.000693	0.007	0.023
DMBX1	0.000667	<0.001	<0.001
MBD2	0.000653	0.016	0.045
HESX1	0.000632	0.002	0.009
PURA	0.000631	0.011	0.033
PARP1	0.000631	0.011	0.034
RCOR1	0.000628	0.011	0.034
ONECUT2	0.000609	0.003	0.012
SCRT1	0.000586	<0.001	<0.001
MYB	0.000586	<0.001	<0.001
ATF7	0.000548	0.007	0.023
ZFP1	0.000539	0.007	0.023
TBPL2	0.000492	0.006	0.021
POU6F1	0.000442	0.001	0.005
BARX1	0.000413	<0.001	<0.001
BARX2	0.000399	0.006	0.02
AHDC1	0.000385	0.004	0.014
SIX3	0.000347	<0.001	0.002
FOXL2	0.000344	<0.001	0.004
OLIG3	0.000337	<0.001	<0.001
KLF14	0.00032	<0.001	<0.001
EMX1	0.000298	0.007	0.024
PRRX1	0.000281	<0.001	<0.001
HOXA13	0.00028	<0.001	<0.001
TLX2	0.000145	<0.001	<0.001
MYOD1	0.000138	<0.001	0.002
POU4F2	7.2e-05	0.009	0.029

B

TF	mean diff-reg stat	p-val	adj p-val
ARID3A	0.0134	<0.001	<0.001
EGR1	0.0112	<0.001	<0.001
ELF1	0.0069	<0.001	<0.001
PRDM11	0.00256	<0.001	<0.001
E2F2	0.00233	<0.001	0.002
ATF3	0.00174	<0.001	<0.001
CRX	0.00172	<0.001	<0.001
SPDEF	0.00163	<0.001	<0.001
HLX	0.0014	<0.001	<0.001
EHF	0.00138	<0.001	<0.001
NR2E3	0.00132	<0.001	<0.001
LIN28B	0.00121	0.002	0.009
EN1	0.0012	<0.001	0.003
HOXA9	0.00118	<0.001	<0.001
CREB1	0.00105	<0.001	0.003
NFIB	0.00101	<0.001	<0.001
ZFP62	0.000916	0.017	0.048
HOXA3	0.000913	<0.001	<0.001
ZKSCAN7	0.000906	<0.001	<0.001
HOXA10	0.000885	<0.001	0.003
GSC	0.00086	<0.001	<0.001
RARA	0.000808	<0.001	0.004
NR1H4	0.000797	<0.001	<0.001
JUN	0.000744	<0.001	0.002
VSX2	0.000596	0.002	0.007
SOX14	0.000596	<0.001	0.003
PITX3	0.00056	<0.001	<0.001
SIX1	0.000557	0.012	0.036
RARB	0.000507	0.003	0.011
NKX2-5	0.000487	0.002	0.007
OTP	0.000481	0.005	0.016
EN2	0.000279	<0.001	0.002
MEF2B	0.000251	0.015	0.043
ISX	0.000158	0.008	0.026
HOXC8	0.000123	<0.001	<0.001

Table S2: *Related to Figure 5. Differentially-regulating transcription-factors inferred from the human embryo data. Transcription factors with significant differential regulation (FDR $p\text{-}val < 0.05$, t-test, Benjamini-Hochberg adjustment) showing increased regulation (hyper-regulation) in (a) epiblast and (b) primitive endoderm cells.*

A

TF	mean diff-reg stat	*p*-val	adj *p*-val
E2F1	0.00783	<0.001	<0.001
ELK1	0.00577	<0.001	<0.001
E2F4	0.00519	<0.001	<0.001
CEBPB	0.00359	<0.001	<0.001
SOX10	0.00358	<0.001	<0.001
BPTF	0.00322	<0.001	<0.001
EHF	0.0021	<0.001	<0.001
ZBTB7B	0.00203	<0.001	<0.001
ARNTL	0.00201	<0.001	<0.001
HINFP	0.00178	<0.001	<0.001
FOXJ2	0.00168	<0.001	<0.001
PBX1	0.00152	<0.001	<0.001
NFIB	0.00142	<0.001	<0.001
ZBTB20	0.00142	<0.001	<0.001
NFATC1	0.00137	<0.001	<0.001
FOXP4	0.00134	<0.001	<0.001
YY1	0.00132	<0.001	<0.001
CLOCK	0.00132	<0.001	<0.001
GRHL1	0.00125	<0.001	0.001
PURA	0.00125	<0.001	<0.001
DLX2	0.00114	<0.001	<0.001
RCOR1	0.00113	<0.001	<0.001
AHDC1	0.00111	<0.001	<0.001
SF1	0.00111	<0.001	<0.001
SATB1	0.00109	<0.001	<0.001
ETV4	0.00103	<0.001	<0.001
TFCP2L1	0.00102	<0.001	<0.001
NFIA	0.000999	<0.001	<0.001
ZKSCAN8	0.000982	<0.001	<0.001
MTF1	0.000889	<0.001	<0.001
NCALD	0.000859	<0.001	<0.001
CHD1	0.000782	<0.001	<0.001
ERF	0.000759	<0.001	<0.001
SPDEF	0.000757	<0.001	0.003
ZBTB7A	0.000747	<0.001	<0.001
HMG20B	0.000744	<0.001	<0.001
MYBL2	0.000718	<0.001	<0.001
FOXM1	0.000703	<0.001	<0.001
SOX5	0.000694	<0.001	<0.001
E2F2	0.000692	<0.001	<0.001
TAF6	0.000681	<0.001	<0.001
ZFP62	0.000664	0.005	0.012
IRF4	0.000658	<0.001	<0.001
NR2C2	0.000658	0.005	0.013
CXXC1	0.000632	<0.001	<0.001
E2F6	0.000598	<0.001	0.003
LCORL	0.000567	<0.001	<0.001
FOXA3	0.000536	<0.001	<0.001
PRDM4	0.000534	<0.001	<0.001
RFX3	0.000497	0.002	0.005
NFIC	0.000496	0.013	0.029
ATF2	0.000411	0.005	0.012
IRF3	0.000405	0.003	0.007
DLX3	0.000396	0.012	0.027
HES7	0.000369	<0.001	<0.001
FUBP1	0.000358	0.009	0.02
SALL2	0.000329	0.001	0.004
PHTF1	0.000314	0.009	0.021
AHCTF1	0.000313	0.011	0.024
HDAC8	0.000309	0.005	0.012
HMG20A	0.000294	0.006	0.014
ERG	0.000268	0.002	0.004
TEF	0.000265	0.002	0.005
ZKSCAN3	0.000259	<0.001	<0.001
HOXC8	0.000253	0.012	0.027
MYB	0.000248	0.002	0.006
SMAD6	0.000227	<0.001	0.002
FOXJ1	0.000193	<0.001	<0.001
FOXL2	0.000179	<0.001	0.002
SPIB	0.000171	<0.001	<0.001
PAX2	0.000141	<0.001	<0.001
ZFP92	0.000139	<0.001	<0.001
FOXP3	0.000129	0.003	0.008
HAND1	0.000128	0.004	0.01
KLF14	0.000106	0.017	0.036

B

TF	mean diff-reg stat	*p*-val	adj *p*-val
BACH1	0.00373	<0.001	<0.001
BCLAF1	0.00363	<0.001	<0.001
FOXC2	0.00281	<0.001	<0.001
MAX	0.00278	<0.001	<0.001
ATF3	0.00278	<0.001	<0.001
ELF1	0.00277	<0.001	<0.001
HDAC2	0.00262	<0.001	<0.001
STAT1	0.00261	<0.001	<0.001
FOXC1	0.00259	<0.001	<0.001
ETS1	0.00246	<0.001	<0.001
SP100	0.00235	<0.001	<0.001
CHURC1	0.00226	<0.001	<0.001
ELK3	0.00212	<0.001	<0.001
NFKB1	0.00201	<0.001	<0.001
ELF3	0.00186	<0.001	<0.001
PRRX2	0.00183	<0.001	<0.001
KLF10	0.00151	<0.001	<0.001
ARID3A	0.00146	0.001	0.003
TAF1	0.00143	<0.001	<0.001
IRF6	0.00141	<0.001	<0.001
SOX4	0.00136	<0.001	<0.001
BCL6	0.00132	<0.001	<0.001
SP1	0.00122	<0.001	<0.001
RELA	0.00109	<0.001	<0.001
HMGA1	0.00109	<0.001	0.001
ID2	0.00109	<0.001	<0.001
IRF2	0.001	<0.001	<0.001
CEBPA	0.000999	<0.001	<0.001
PDLIM5	0.000987	<0.001	<0.001
MEF2A	0.000924	<0.001	<0.001
MTA3	0.000789	<0.001	<0.001
ZFP57	0.000773	<0.001	<0.001
YOD1	0.000753	<0.001	<0.001
ARID5A	0.000745	0.003	0.009
IRF1	0.000726	0.003	0.007
RARB	0.000724	<0.001	<0.001
PHF1	0.000684	<0.001	<0.001
ELK4	0.000639	<0.001	<0.001
ZSCAN2	0.000593	<0.001	<0.001
MLX	0.000567	<0.001	<0.001
HBP1	0.000552	<0.001	<0.001
VEZF1	0.000537	<0.001	0.002
MSX2	0.000486	<0.001	<0.001
KLF13	0.000484	0.013	0.027
SP2	0.000474	<0.001	<0.001
HSF1	0.000437	0.005	0.013
HOXB2	0.000427	<0.001	<0.001
IRF9	0.000421	0.004	0.01
ARID5B	0.000405	0.008	0.019
MYBL1	0.000392	0.013	0.028
CNOT3	0.000358	0.018	0.038
MYC	0.000348	0.005	0.013
RXRA	0.000347	0.01	0.022
FOXJ3	0.000346	<0.001	0.002
MEF2B	0.000344	<0.001	<0.001
STAT4	0.000306	0.002	0.005
FOXO3	0.000292	0.019	0.04
ZFHX3	0.000255	0.022	0.047
NKX2-5	0.000207	0.007	0.016
ZIC1	0.000187	<0.001	<0.001
FOXD3	0.00015	<0.001	<0.001
CDX1	0.000148	0.004	0.011

Table S3: *Related to Figure 7. Differentially-regulating transcription factors inferred from the breast-cancer at-risk data, in luminal progenitor cells. Transcription factors with significant differential regulation (FDR* $p\text{-}val < 0.05$*, t-test, Benjamini-Hochberg adjustment) in luminal progenitor cells that are (a) hypo-regulating and (b) hyper-regulating in BRCA1/2 mutation carriers (BRCAmut) compared to BRCA1/2 wild-type (WT).*

A

TF	mean diff-reg stat	*p*-val	adj *p*-val
BPTF	0.0133	<0.001	<0.001
ELF1	0.0125	<0.001	<0.001
FOXA1	0.00506	<0.001	<0.001
ARID3A	0.00305	<0.001	<0.001
PURA	0.00299	<0.001	<0.001
E2F1	0.00299	<0.001	<0.001
CEBPZ	0.00287	<0.001	<0.001
YY1	0.00267	<0.001	<0.001
SPDEF	0.00251	<0.001	<0.001
ZBTB20	0.00245	<0.001	<0.001
CTCF	0.00226	<0.001	<0.001
FUBP1	0.0022	<0.001	<0.001
CLOCK	0.00217	<0.001	<0.001
NFIA	0.00205	<0.001	<0.001
SF1	0.00202	<0.001	<0.001
ZBTB7A	0.00199	<0.001	<0.001
PBX1	0.00163	<0.001	<0.001
NFIB	0.00153	<0.001	<0.001
CHD1	0.00152	<0.001	<0.001
FOXM1	0.00147	<0.001	<0.001
E2F4	0.00126	0.001	0.003
RCOR1	0.00122	<0.001	<0.001
HINFP	0.00111	<0.001	<0.001
MTF1	0.00111	<0.001	<0.001
ARNTL	0.0011	<0.001	<0.001
RFX3	0.00106	<0.001	<0.001
KLF5	0.00104	<0.001	<0.001
GRHL1	0.00103	0.002	0.004
MYB	0.00102	<0.001	<0.001
GMEB1	0.000936	<0.001	<0.001
NCALD	0.000913	<0.001	<0.001
E2F2	0.000899	<0.001	<0.001
NFKB1	0.000879	<0.001	<0.001
ZSCAN29	0.00087	<0.001	<0.001
ZBTB43	0.000781	<0.001	<0.001
FOXJ2	0.000744	0.003	0.006
MSX2	0.000639	<0.001	0.002
GATA3	0.00058	<0.001	<0.001
AHCTF1	0.000574	<0.001	<0.001
HMG20B	0.000567	0.002	0.005
AR	0.000549	<0.001	<0.001
SOX5	0.00053	<0.001	<0.001
ZFP3	0.000523	<0.001	<0.001
EBF1	0.000507	<0.001	<0.001
MYBL2	0.000481	<0.001	<0.001
RFXANK	0.000451	0.001	0.003
NR2E3	0.000448	<0.001	<0.001
PRRX1	0.000446	<0.001	<0.001
HOXA10	0.000444	<0.001	0.001
FOXP3	0.000429	<0.001	<0.001
ERF	0.00042	0.003	0.007
ALX4	0.000374	<0.001	<0.001
FOXO3	0.000368	0.024	0.046
ZBTB2	0.000366	0.011	0.023
HOXD10	0.000328	<0.001	<0.001
HDAC8	0.000324	0.003	0.006
RARB	0.000299	<0.001	<0.001
FOXA3	0.000299	0.009	0.019
MSX1	0.000296	<0.001	<0.001
FOXJ1	0.000265	<0.001	0.002
POU3F1	0.000259	<0.001	<0.001
HNF4G	0.000225	0.01	0.021
SOX10	0.000222	0.001	0.003
SOX18	0.000206	<0.001	<0.001

B

TF	mean diff-reg stat	*p*-val	adj *p*-val
AHDC1	0.00463	<0.001	<0.001
DRAP1	0.00407	<0.001	<0.001
BCL6	0.00401	<0.001	<0.001
CHURC1	0.00376	<0.001	<0.001
ATF3	0.00343	<0.001	<0.001
SOX4	0.00262	<0.001	<0.001
HMGA1	0.0025	<0.001	<0.001
ETS1	0.00208	<0.001	<0.001
SP1	0.00202	<0.001	<0.001
HDAC2	0.00201	<0.001	<0.001
TAF1	0.0019	<0.001	<0.001
BHLHE40	0.00187	<0.001	<0.001
MYC	0.00176	<0.001	<0.001
PHF1	0.00173	<0.001	<0.001
PDLIM5	0.00168	<0.001	<0.001
SP2	0.00168	<0.001	<0.001
MAZ	0.00164	<0.001	<0.001
HOXB2	0.00161	<0.001	<0.001
ELF3	0.00151	<0.001	<0.001
ATF7	0.0015	<0.001	<0.001
BACH1	0.00143	0.009	0.019
ZBTB14	0.00111	0.002	0.006
KLF11	0.00111	<0.001	<0.001
AHR	0.00111	<0.001	<0.001
MEF2D	0.0011	<0.001	<0.001
PRDM10	0.00105	<0.001	<0.001
ARNT	0.00104	<0.001	<0.001
FOXC1	0.00104	<0.001	<0.001
SP100	0.00101	<0.001	<0.001
HOXC10	0.00101	<0.001	<0.001
IRF2	0.000992	<0.001	<0.001
LEF1	0.000986	<0.001	<0.001
EHF	0.000973	0.012	0.023
ZBTB41	0.00094	<0.001	<0.001
KLF10	0.000934	<0.001	<0.001
ARID5A	0.00093	<0.001	0.002
ZSCAN2	0.000872	<0.001	<0.001
CCDC160	0.000839	0.002	0.004
SOX11	0.000834	<0.001	<0.001
STAT1	0.000768	0.013	0.025
MEF2A	0.000744	<0.001	<0.001
YOD1	0.000733	<0.001	<0.001
GMEB2	0.000732	<0.001	<0.001
HOXB7	0.000707	<0.001	<0.001
NFE2L1	0.000685	0.01	0.021
RXRA	0.000664	<0.001	<0.001
ATF1	0.000662	0.002	0.006
NR2C2	0.000659	<0.001	0.002
ARID3B	0.00049	0.005	0.012
CEBPA	0.000485	<0.001	0.002
ELK3	0.000467	0.003	0.008
TEF	0.000464	0.005	0.01
FOSL2	0.000461	<0.001	0.001
IKZF3	0.00046	<0.001	<0.001
HBP1	0.000408	0.006	0.014
RELA	0.000398	0.013	0.025
NMRAL1	0.000392	0.018	0.035
EN1	0.000372	<0.001	<0.001
HES7	0.000363	<0.001	<0.001
CCNT2	0.000361	0.012	0.024
FOXN3	0.000344	0.025	0.047
ZFP37	0.000321	0.007	0.016
NF1	0.000321	0.008	0.016
BACH2	0.000314	0.007	0.016
SNAPC4	0.000314	0.004	0.01
ABL1	0.000275	0.003	0.007
MNX1	0.000259	<0.001	0.002
ZBTB12	0.000233	0.01	0.021
PITX3	0.000224	<0.001	<0.001
SP5	0.000179	0.006	0.014
ARX	0.000162	0.016	0.03
HOXB9	0.000158	<0.001	<0.001
HOXC13	0.000149	0.005	0.011
HOXA11	0.000114	0.003	0.007

Table S4: *Related to Figure 7. Differentially-regulating transcription factors inferred from the breast-cancer at-risk data, in luminal mature cells. Transcription factors with significant differential regulation (FDR* $p\text{-}val < 0.05$*, t-test, Benjamini-Hochberg adjustment) in luminal mature cells that are (a) hypo-regulating and (b) hyper-regulating in BRCA1/2 mutation carriers (BRCAmut) compared to BRCA1/2 wild-type (WT).*

A

TF	mean diff-reg stat	*p*-val	adj *p*-val
EGR1	0.0209	<0.001	<0.001
BPTF	0.0122	<0.001	<0.001
KLF4	0.00629	<0.001	<0.001
FOXC1	0.0057	<0.001	<0.001
ELF1	0.00556	<0.001	<0.001
ZBTB20	0.00526	<0.001	<0.001
NFIA	0.00453	<0.001	<0.001
KLF16	0.00443	<0.001	<0.001
CHD1	0.00383	<0.001	<0.001
BACH1	0.00365	<0.001	<0.001
KLF5	0.00338	<0.001	<0.001
ATF3	0.00331	<0.001	<0.001
ZBTB14	0.0031	<0.001	<0.001
SOX11	0.00269	<0.001	<0.001
PBX1	0.00236	<0.001	<0.001
BHLHE40	0.00235	<0.001	<0.001
GLI3	0.00219	<0.001	<0.001
RCOR1	0.00217	<0.001	<0.001
EHF	0.00217	<0.001	<0.001
KLF13	0.00213	<0.001	<0.001
EGR2	0.00209	<0.001	<0.001
YY1	0.00207	<0.001	<0.001
ERF	0.00193	<0.001	<0.001
PURA	0.00186	<0.001	<0.001
ELF3	0.00179	<0.001	<0.001
SOX5	0.00176	<0.001	<0.001
ZBTB7A	0.00174	<0.001	<0.001
DLX2	0.00161	<0.001	<0.001
ZBTB11	0.00157	<0.001	<0.001
AHCTF1	0.00157	<0.001	<0.001
MAFB	0.00155	<0.001	<0.001
ATF2	0.0015	<0.001	<0.001
DLX3	0.00148	<0.001	<0.001
FOXO1	0.00146	<0.001	<0.001
SOX9	0.00145	<0.001	<0.001
POU3F1	0.00141	<0.001	<0.001
HIC1	0.0014	<0.001	<0.001
E2F1	0.00134	<0.001	<0.001
ZBTB12	0.00132	<0.001	<0.001
SPDEF	0.00131	<0.001	<0.001
ALX4	0.0013	<0.001	<0.001
ELF2	0.00129	<0.001	<0.001
ALX3	0.00111	<0.001	<0.001
CEBPA	0.00108	<0.001	<0.001
ID2	0.00104	<0.001	<0.001
BCL6	0.00101	<0.001	<0.001
SATB1	0.001	<0.001	<0.001
HIC2	0.000937	<0.001	<0.001
RBAK	0.000929	<0.001	<0.001
NCALD	0.00089	<0.001	<0.001
TCFL5	0.000862	<0.001	<0.001
EGR4	0.000854	<0.001	<0.001
PRDM10	0.000816	<0.001	<0.001
SALL2	0.000815	<0.001	<0.001
EN1	0.000741	<0.001	<0.001
ASCL1	0.000733	<0.001	<0.001
RXRA	0.000732	<0.001	<0.001
REST	0.000727	<0.001	<0.001
POU2F1	0.000695	0.003	0.006
KLF2	0.000633	<0.001	<0.001
MSX2	0.000631	0.001	0.002
KLF10	0.00063	<0.001	0.002
FOXL2	0.00061	<0.001	<0.001
CEBPZ	0.00061	0.024	0.035
AHR	0.000607	0.012	0.019
IRF4	0.000603	<0.001	<0.001
ZBTB33	0.000597	0.012	0.018
MYBL1	0.00057	<0.001	<0.001
PLAG1	0.000544	<0.001	<0.001
SOX10	0.000491	<0.001	<0.001
MEF2D	0.000483	0.002	0.004
PRDM5	0.00047	0.003	0.005
ZBTB41	0.000464	0.002	0.003
VEZF1	0.000446	0.016	0.024
LCORL	0.00042	0.007	0.012
FOXA1	0.000413	<0.001	<0.001
CRX	0.000407	<0.001	0.001
EBF1	0.000406	<0.001	<0.001
ARX	0.00037	<0.001	<0.001
MYB	0.000355	<0.001	<0.001
HLX	0.000352	0.003	0.005
HOXC10	0.000351	0.012	0.019
BRCA1	0.00035	0.004	0.007
MNX1	0.000327	<0.001	<0.001
SIX1	0.000314	<0.001	<0.001
MEF2C	0.00031	0.009	0.015
GLI1	0.000302	<0.001	<0.001
NKX2-5	0.000286	0.004	0.007
SPI1	0.000268	<0.001	<0.001
MSX1	0.000266	0.016	0.024
GRHL1	0.000261	0.023	0.034
NR2E3	0.000258	<0.001	<0.001
HOXB7	0.000242	<0.001	<0.001
KLF17	0.000236	<0.001	<0.001
CCDC160	0.000229	<0.001	<0.001
HOXD9	0.000214	0.003	0.005
EN2	0.000196	0.003	0.005
E2F2	0.000189	<0.001	<0.001
SIX3	0.000178	0.006	0.009
MYBL2	0.000175	0.014	0.021
SOX6	0.000157	<0.001	0.001
SOX18	0.000154	0.01	0.016
OTX1	0.000154	<0.001	<0.001
ZFP92	0.000148	0.013	0.02
POU3F2	0.000147	0.003	0.005
PAX2	0.000128	<0.001	<0.001
FOXD3	0.000127	<0.001	<0.001
DMRT2	0.000124	<0.001	<0.001

B

TF	mean diff-reg stat	*p*-val	adj *p*-val
E2F4	0.0171	<0.001	<0.001
ELK1	0.00997	<0.001	<0.001
HDAC2	0.00557	<0.001	<0.001
MAX	0.00422	<0.001	<0.001
DRAP1	0.00385	<0.001	<0.001
STAT1	0.00368	<0.001	<0.001
NFIB	0.00319	<0.001	<0.001
HMGA1	0.00292	<0.001	<0.001
MAZ	0.0029	<0.001	<0.001
HINFP	0.00236	<0.001	<0.001
CLOCK	0.00221	<0.001	<0.001
DEAF1	0.00215	<0.001	<0.001
TBP	0.00211	<0.001	<0.001
PARP1	0.00204	<0.001	<0.001
CNOT3	0.00198	<0.001	<0.001
HOXA9	0.00193	<0.001	<0.001
TAF6	0.00187	<0.001	<0.001
KLF6	0.00172	<0.001	<0.001
NRF1	0.00167	<0.001	<0.001
ARNT	0.00162	<0.001	<0.001
ATF1	0.0016	<0.001	<0.001
ARID5A	0.00158	<0.001	<0.001
ZFP62	0.00148	<0.001	<0.001
SOX4	0.00147	0.001	0.002
FOXC2	0.00133	<0.001	<0.001
NFYC	0.00131	<0.001	<0.001
CEBPD	0.0013	<0.001	<0.001
GMEB1	0.00121	<0.001	<0.001
CXXC1	0.00119	<0.001	<0.001
IRF2	0.00117	<0.001	<0.001
TEAD3	0.00117	<0.001	<0.001
HMG20B	0.00112	<0.001	<0.001
CHURC1	0.00109	<0.001	<0.001
CTCF	0.00106	0.006	0.01
E2F6	0.00106	<0.001	<0.001
ELK3	0.00101	<0.001	<0.001
NFIC	0.001	<0.001	<0.001
IRF3	0.000984	<0.001	<0.001
NR2C2	0.000961	<0.001	0.001
STAT3	0.000922	0.007	0.012
PHF1	0.000885	<0.001	<0.001
MYPOP	0.000868	<0.001	<0.001
HOMEZ	0.000817	<0.001	<0.001
YEATS4	0.000798	<0.001	<0.001
SREBF2	0.00079	<0.001	<0.001
TEAD4	0.000778	<0.001	<0.001
ARNTL	0.000734	0.01	0.016
MYC	0.000731	0.002	0.003
FUBP1	0.000724	<0.001	0.002
DDX20	0.000699	<0.001	<0.001
PHTF1	0.000659	<0.001	<0.001
MEF2B	0.000658	<0.001	<0.001
NMRAL1	0.000654	<0.001	<0.001
GMEB2	0.000641	<0.001	<0.001
IRF7	0.000623	<0.001	<0.001
SF1	0.000606	0.006	0.01
MEF2A	0.000603	0.02	0.03
CUX1	0.000573	0.032	0.046
MTF1	0.000568	<0.001	0.001
TEAD1	0.000562	0.01	0.016
ATF7	0.000528	0.002	0.004
CREB1	0.000482	0.009	0.015
HDAC8	0.000442	<0.001	<0.001
BRF1	0.000439	0.022	0.033
PRRX2	0.000344	0.014	0.021
HOXC8	0.000237	0.028	0.041
ZSCAN20	0.000224	0.002	0.004

Table S5: *Related to Figure 7. Differentially-regulating transcription factors inferred from the breast-cancer at-risk data, in basal cells. Transcription factors with significant differential regulation (FDR* p-val $<$ 0.05*, t-test, Benjamini-Hochberg adjustment) in basal cells that are (a) hypo-regulating and (b) hyper-regulating in BRCA1/2 mutation carriers (BRCAmut) compared to BRCA1/2 wild-type (WT).*

A

TF	mean diff-reg stat	p-val	adj p-val
CLOCK	0.0054	<0.001	<0.001
STAT1	0.00503	<0.001	<0.001
BACH1	0.00389	<0.001	<0.001
HDAC2	0.00346	<0.001	<0.001
CTCF	0.00326	<0.001	<0.001
KLF13	0.00242	<0.001	<0.001
ARID3A	0.0024	<0.001	<0.001
IRF4	0.00209	<0.001	<0.001
BPTF	0.00209	<0.001	<0.001
RBAK	0.00205	<0.001	<0.001
PARP1	0.00201	<0.001	<0.001
CEBPA	0.00188	<0.001	<0.001
PRRX2	0.00181	<0.001	<0.001
SP1	0.00166	<0.001	<0.001
CDX1	0.00161	<0.001	<0.001
FUBP1	0.00159	<0.001	<0.001
EGR2	0.00157	<0.001	<0.001
EHF	0.00154	<0.001	<0.001
FOXM1	0.00149	<0.001	<0.001
TOPORS	0.00142	<0.001	<0.001
ZBTB7A	0.00137	<0.001	<0.001
GMEB1	0.00136	<0.001	<0.001
E2F3	0.00134	<0.001	<0.001
SP100	0.00131	<0.001	<0.001
GABPA	0.00131	<0.001	<0.001
RCOR1	0.00127	<0.001	<0.001
ELK4	0.00112	<0.001	<0.001
NFATC4	0.00111	<0.001	<0.001
STAT4	0.00111	<0.001	<0.001
FOXJ2	0.00109	<0.001	<0.001
ERF	0.00103	<0.001	<0.001
TAF1	0.00102	<0.001	<0.001
TCFL5	0.000987	<0.001	<0.001
NCALD	0.000986	<0.001	<0.001
NFE2L1	0.000889	<0.001	<0.001
ELK3	0.000882	<0.001	<0.001
E2F2	0.000857	<0.001	<0.001
CEBPZ	0.000835	<0.001	<0.001
RFX2	0.000833	<0.001	<0.001
VEZF1	8e-04	<0.001	<0.001
DLX3	0.000798	<0.001	<0.001
NR2C2	0.000796	<0.001	<0.001
NFATC3	0.000788	<0.001	<0.001
ZSCAN29	0.000778	<0.001	<0.001
HIC2	0.000771	<0.001	<0.001
FOXL2	0.000769	<0.001	<0.001
CRX	0.00076	<0.001	<0.001
AHCTF1	0.000741	<0.001	<0.001
ETS1	0.000724	0.012	0.022
NR4A2	0.000719	<0.001	<0.001
BACH2	0.000719	<0.001	0.001
POU2F1	0.000711	<0.001	<0.001
SATB1	0.00071	<0.001	<0.001
ZBTB37	0.000688	<0.001	<0.001
CNOT3	0.000677	<0.001	0.002
BRCA1	0.000658	<0.001	<0.001
TBP	0.000657	0.002	0.004
ZKSCAN8	0.00065	<0.001	<0.001
ARID5A	0.000649	<0.001	<0.001
ALX3	6e-04	0.001	0.003
E2F8	0.000591	<0.001	<0.001
KLF16	0.000589	<0.001	<0.001
GLI3	0.000535	0.002	0.005
ZBTB11	0.000533	0.004	0.009
ZEB1	0.000531	0.006	0.012
ARID3B	0.000519	<0.001	<0.001
SIX3	5e-04	<0.001	<0.001
GMEB2	0.000489	0.001	0.003
ASCL1	0.000474	<0.001	<0.001
ATF7	0.000459	0.003	0.007
SPDEF	0.000425	<0.001	<0.001
KLF2	0.000425	0.006	0.012
ZFP92	0.000396	<0.001	<0.001
FOXC2	0.000377	0.009	0.017
OTX1	0.000371	<0.001	<0.001
ZFP3	0.000369	<0.001	<0.001
HOXA13	0.000339	<0.001	0.002
IKZF1	0.000332	<0.001	<0.001
NR2E3	0.00033	<0.001	<0.001
PURG	0.000286	0.006	0.011
FOXP3	0.00028	0.005	0.01
BCL6B	0.000278	<0.001	<0.001
IKZF3	0.000255	0.009	0.016
IRF6	0.000214	0.005	0.01
GATA3	0.000193	0.01	0.018
PATZ1	0.000191	0.02	0.035
MYBL2	0.000181	<0.001	<0.001
MYB	0.000145	0.029	0.05
SOX11	0.000139	0.012	0.021
CCDC160	0.000139	0.01	0.018
POU4F3	0.000127	<0.001	<0.001
PAX2	0.000126	<0.001	<0.001
NKX2-5	9.87e-05	0.003	0.007
ARX	8.32e-05	<0.001	<0.001

B

TF	mean diff-reg stat	p-val	adj p-val
EGR1	0.0122	<0.001	<0.001
CHURC1	0.00335	<0.001	<0.001
ELF1	0.00319	<0.001	<0.001
E2F4	0.00284	<0.001	<0.001
ELK1	0.00261	<0.001	<0.001
ARNTL	0.00245	<0.001	<0.001
ATF2	0.0021	<0.001	<0.001
HMGA1	0.00192	<0.001	<0.001
CEBPB	0.00183	<0.001	<0.001
DEAF1	0.00176	<0.001	<0.001
BCLAF1	0.00176	<0.001	0.002
BCL6	0.00171	<0.001	<0.001
KLF5	0.00159	<0.001	<0.001
PDLIM5	0.00143	<0.001	<0.001
ZBTB14	0.0014	<0.001	<0.001
ARNT	0.00138	<0.001	<0.001
MAX	0.00133	<0.001	<0.001
DRAP1	0.00129	<0.001	<0.001
STAT5A	0.00115	<0.001	<0.001
IRF7	0.00114	<0.001	<0.001
MAZ	0.0011	0.001	0.002
STAT3	0.0011	<0.001	<0.001
KLF11	0.000963	<0.001	<0.001
ZBTB2	0.000956	<0.001	<0.001
TWIST1	0.000894	<0.001	<0.001
KLF9	0.000885	<0.001	<0.001
MEF2B	0.000854	<0.001	<0.001
LEF1	0.000847	<0.001	<0.001
HSF1	0.00078	<0.001	<0.001
RAB18	0.000747	<0.001	<0.001
KLF10	0.000722	0.002	0.004
HOXD10	0.000708	<0.001	<0.001
PRRX1	0.000683	<0.001	0.001
SF1	0.000668	<0.001	<0.001
RARB	0.000657	<0.001	<0.001
SREBF2	0.000654	<0.001	<0.001
SOX4	0.000628	<0.001	<0.001
ILF2	0.000622	<0.001	<0.001
ID2	0.000606	0.002	0.005
MAFB	0.000538	<0.001	<0.001
TEAD4	0.000531	<0.001	<0.001
YY1	0.000525	0.004	0.009
IRF2	0.000508	0.023	0.039
MSX2	0.000489	<0.001	<0.001
YOD1	0.00048	<0.001	<0.001
CXXC1	0.000477	0.007	0.013
KLF15	0.000455	<0.001	<0.001
POU3F1	0.000441	<0.001	<0.001
MSX1	0.000408	0.016	0.029
MEF2D	0.00039	0.005	0.01
IRF1	0.000379	0.028	0.049
DMRT2	0.000369	0.008	0.015
HOXC8	0.000346	0.013	0.023
ZBTB26	0.00033	0.002	0.004
GRHL1	0.000327	0.004	0.008
HOXB2	0.000284	0.01	0.018
MEF2C	0.000284	0.017	0.03
FOXO6	0.000253	0.003	0.006
HOXD3	0.000245	0.006	0.012
HOXA3	0.000228	0.006	0.012
EN2	0.000213	0.02	0.035
MECOM	0.000209	0.005	0.01
POU3F2	2e-04	<0.001	0.002
SOX18	0.000181	0.012	0.022
E2F7	0.000164	0.002	0.004
FOXD3	0.000164	<0.001	<0.001
CEBPE	0.000161	0.002	0.005
ISL1	0.000153	<0.001	0.002

Table S6: *Related to Figure 7. Differentially-regulating transcription factors inferred from the breast-cancer at-risk data, in fibroblasts. Transcription factors with significant differential regulation (FDR* $p\text{-}val < 0.05$*, t-test, Benjamini-Hochberg adjustment) in fibroblasts (type 1) that are (a) hypo-regulating and (b) hyper-regulating in BRCA1/2 mutation carriers (BRCAmut) compared to BRCA1/2 wild-type (WT).*

A

TF	mean diff-reg stat	*p*-val	adj *p*-val
BCLAF1	0.00631	<0.001	<0.001
BPTF	0.00453	<0.001	<0.001
ELF1	0.00374	<0.001	<0.001
KLF4	0.00355	<0.001	<0.001
CLOCK	0.00275	<0.001	<0.001
PURA	0.00263	<0.001	<0.001
CTCF	0.00233	<0.001	<0.001
FOXC1	0.00204	<0.001	<0.001
CEBPZ	0.0019	<0.001	<0.001
ZBTB14	0.00189	<0.001	<0.001
EHF	0.00182	<0.001	<0.001
STAT1	0.00166	<0.001	<0.001
ARID3A	0.00165	0.002	0.005
CEBPA	0.00157	<0.001	<0.001
DLX2	0.00155	<0.001	<0.001
PARP1	0.00142	<0.001	<0.001
E2F1	0.00134	<0.001	<0.001
PRRX2	0.00132	<0.001	<0.001
MAZ	0.00131	<0.001	0.001
ATF3	0.00122	0.005	0.011
DLX3	0.0012	<0.001	<0.001
ZBTB20	0.0012	<0.001	<0.001
CUX1	0.0012	<0.001	<0.001
SF1	0.00114	<0.001	<0.001
NFATC1	0.00112	<0.001	<0.001
CDX1	0.0011	<0.001	<0.001
EGR2	0.00108	<0.001	<0.001
ELF3	0.00105	<0.001	<0.001
KLF2	0.000999	<0.001	<0.001
IRF3	0.000943	<0.001	<0.001
KLF13	0.000922	0.001	0.003
CREB1	0.000909	<0.001	<0.001
EGR4	0.000906	<0.001	<0.001
CHD1	0.000896	<0.001	<0.001
TOPORS	0.000781	<0.001	<0.001
NR4A2	0.000743	<0.001	<0.001
ELK3	0.000715	0.002	0.005
CDC5L	0.00071	0.007	0.015
YY1	0.000684	0.002	0.005
HOXC8	0.000642	<0.001	<0.001
STAT4	0.000624	0.001	0.004
ABL1	0.000597	<0.001	0.002
ALX4	0.000593	<0.001	<0.001
ZSCAN29	0.000561	<0.001	<0.001
ZBTB11	0.000559	0.001	0.003
KLF6	0.000545	0.005	0.012
SIX3	0.000538	<0.001	<0.001
OVOL1	0.00051	<0.001	<0.001
SOX18	0.000489	<0.001	<0.001
E2F2	0.000466	<0.001	<0.001
DLX4	0.000461	<0.001	<0.001
HOXA13	0.000455	<0.001	<0.001
ZBTB7A	0.000455	0.006	0.014
ZBTB2	0.000443	0.001	0.003
VEZF1	0.000443	0.019	0.037
ID2	0.000426	0.017	0.034
HMG20A	0.000419	0.002	0.005
ZIC1	0.000418	<0.001	<0.001
ZBTB12	0.000416	<0.001	<0.001
HOXA10	0.000405	0.008	0.016
FOXC2	0.000394	0.002	0.004
NFATC3	0.000389	<0.001	<0.001
DBX2	0.000378	<0.001	<0.001
NCALD	0.000376	<0.001	<0.001
FOXJ3	0.000364	0.006	0.013
HOXA11	0.000337	<0.001	<0.001
SIX5	0.000337	0.006	0.014
HES7	0.000318	0.012	0.024
BRCA1	0.000302	0.003	0.007
IKZF1	0.000302	<0.001	<0.001
IRF6	0.00029	0.001	0.003
FOXA1	0.000289	0.019	0.037
SOX11	0.000276	<0.001	<0.001
OVOL2	0.000246	<0.001	<0.001
BCL6B	0.000242	<0.001	0.003
PURG	0.000232	0.002	0.006
DMRT2	0.000182	0.015	0.03
BARX2	0.000141	0.024	0.047
NKX2-5	0.000122	<0.001	<0.001
MECOM	0.000101	0.024	0.046
KLF17	9.16e-05	0.015	0.03
PAX2	8.86e-05	0.023	0.045
HAND1	6.32e-05	0.025	0.047
OTX1	5.86e-05	0.004	0.009

B

TF	mean diff-reg stat	*p*-val	adj *p*-val
ELK1	0.00742	<0.001	<0.001
E2F4	0.0053	<0.001	<0.001
MAX	0.00325	<0.001	<0.001
KLF9	0.00277	<0.001	<0.001
ARNT	0.00276	<0.001	<0.001
DRAP1	0.00272	<0.001	<0.001
BCL6	0.00261	<0.001	<0.001
CEBPB	0.0024	<0.001	<0.001
CHURC1	0.00194	<0.001	<0.001
MEF2D	0.00179	<0.001	<0.001
ZFHX3	0.00175	<0.001	<0.001
PDLIM5	0.00167	<0.001	<0.001
MEF2A	0.00161	<0.001	<0.001
ARNTL	0.00151	<0.001	<0.001
BACH1	0.00145	0.006	0.014
SP1	0.00143	<0.001	<0.001
SREBF2	0.00141	<0.001	<0.001
HDAC2	0.00122	<0.001	<0.001
MSC	0.00121	<0.001	<0.001
ETS1	0.00119	<0.001	<0.001
NRF1	0.00111	<0.001	<0.001
AHR	0.00107	<0.001	<0.001
HSF1	0.00105	<0.001	<0.001
ILF2	0.000993	<0.001	<0.001
TBP	0.000991	<0.001	0.001
ATF2	0.000987	<0.001	<0.001
IRF9	0.000986	<0.001	<0.001
MECP2	0.000945	<0.001	<0.001
STAT5A	0.000907	<0.001	<0.001
LEF1	0.00088	<0.001	<0.001
KLF5	0.000843	<0.001	0.002
CNOT3	0.000838	<0.001	<0.001
TAF1	0.000833	<0.001	<0.001
SP2	0.000822	0.002	0.005
KLF11	0.000804	<0.001	0.001
ZBTB41	0.000737	<0.001	<0.001
YOD1	0.000715	<0.001	<0.001
MEF2B	7e-04	<0.001	<0.001
NFATC4	0.000682	<0.001	<0.001
NR2C2	0.000676	<0.001	0.001
E2F6	0.000664	<0.001	<0.001
GMEB2	0.000636	<0.001	<0.001
STAT3	0.000636	0.001	0.004
RAB18	0.00063	<0.001	<0.001
PBX1	0.000626	<0.001	0.002
BACH2	0.000604	0.002	0.005
KLF15	0.000595	<0.001	<0.001
EN2	0.000592	<0.001	<0.001
ATF7	0.000563	<0.001	0.001
POU2F1	0.000543	0.009	0.019
TFE3	0.00047	0.002	0.005
NRL	0.000441	<0.001	<0.001
ELK4	0.000416	0.013	0.026
TWIST1	0.000396	0.017	0.035
SNAPC5	0.000373	0.003	0.007
ASCL1	0.00037	<0.001	<0.001
HLX	0.000361	0.012	0.024
POU3F1	0.000319	0.007	0.016
HOXD10	0.000306	0.006	0.014
ZFP92	0.000257	0.022	0.044
MYB	0.00025	0.007	0.016

Table S7: *Related to Figure 7. Differentially-regulating transcription factors inferred from the breast-cancer at-risk data, in fibroblast cells. Transcription factors with significant differential regulation (FDR* $p\text{-}val < 0.05$*, t-test, Benjamini-Hochberg adjustment) in fibroblasts (type 2) that are (a) hypo-regulating and (b) hyper-regulating in BRCA1/2 mutation carriers (BRCAmut) compared to BRCA1/2 wild-type (WT).*

	HR (95%CI)	*p*-value
cg10020083 methylation	0.764 (0.395 - 1.48)	0.423
Age (years)	1.53 (1.04 - 2.23)	0.029
AJCC Stage (III-IV vs. I-II)	2.99 (1.37 - 6.52)	0.006
Fibroblast fraction	0.37 (0.006 - 24.2)	0.641
Fat cell fraction	3.2 (0.1 - 102)	0.511
Immune cell fraction	1.1 (0.061 - 19.7)	0.948

	HR (95%CI)	*p*-value
cg23403350 methylation	1.59 (1.2 - 2.12)	0.001
Age (years)	1.65 (1.12 - 2.41)	0.01
AJCC Stage (III-IV vs. I-II)	3.83 (1.72 - 8.54)	0.001
Fibroblast fraction	0.239 (0.004 - 15.6)	0.502
Fat cell fraction	1.19 (0.037 - 38.7)	0.921
Immune cell fraction	0.744 (0.04 - 13.8)	0.842

	HR (95%CI)	*p*-value
cg04110559 methylation	1.67 (1.07 - 2.6)	0.023
Age (years)	1.64 (1.12 - 2.41)	0.011
AJCC Stage (III-IV vs. I-II)	4.02 (1.75 - 9.24)	0.001
Fibroblast fraction	0.206 (0.003 - 12.2)	0.448
Fat cell fraction	1.69 (0.06 - 47)	0.758
Immune cell fraction	0.814 (0.043 - 15.4)	0.891

Table S8: *Related to Figure 7. Survival analyses (Cox regression) showing association of CpG methylation level with patient survival outcome in the TCGA BRCA dataset [12] for the 3 CpGs annotated to the PRR7 promoter (Figure 7) on the Illumina 450K DNAme microarray, adjusted for confounding by cell-type heterogeneity and clinical covariates.*

E6.10.1046	E6.17.1588	E6.9.1024	E7.17.1348
E6.10.1048	E6.17.1611	E7.10.760	E7.17.1353
E6.10.1049	E6.17.1612	E7.10.761	E7.6.262
E6.10.1050	E6.17.1617	E7.10.762	E7.6.264
E6.10.1051	E6.22.1852	E7.10.764	E7.8.311
E6.10.1052	E6.22.1866	E7.10.766	E7.8.312
E6.10.1055	E6.8.791	E7.10.768	E7.8.317
E6.10.1056	E6.8.792	E7.12.858	E7.8.318
E6.10.1057	E6.8.794	E7.12.861	E7.8.329
E6.10.1058	E6.8.801	E7.12.869	E7.8.333
E6.12.1274	E6.8.802	E7.12.871	E7.8.343
E6.12.1289	E6.8.813	E7.14.895	E7.9.547
E6.13.1378	E6.8.815	E7.17.1334	E7.9.554
E6.13.1381	E6.8.816	E7.17.1335	E7.9.556
E6.13.1389	E6.8.820	E7.17.1342	E7.9.562
E6.16.1501	E6.8.821	E7.17.1346	E7.9.569
E6.17.1586	E6.9.1022	E7.17.1347	E7.9.571

Table S9: *Related to Figures 4 and 5. The 68 epiblast cells identified previously [2] and used in this work, identified as cluster . The listed cell IDs correspond to the IDs given in the original study that generated these data [3].*

E5.12.1038	E5.14.1812	E5.37.3243	E6.10.1043
E6.10.1044	E6.10.1045	E6.10.1060	E6.10.1061
E6.12.1273	E6.12.1279	E6.12.1281	E6.13.1382
E6.18.1641	E7.10.767	E7.11.841	E7.11.842
E7.11.843	E7.11.845	E7.11.847	E7.11.855
E7.12.857	E7.12.866	E7.12.872	E7.13.882
E7.E7.13.883	E7.13.884	E7.13.892	E7.14.894
E7.14.904	E7.14.905	E7.14.906	E7.14.908
E7.16.1135	E7.16.1169	E7.8.327	E7.9.539
E7.9.550			

Table S10: *Related to Figures 4 and 5. The 37 PrE (primitive endoderm) cells identified as cluster 4 in Figure S2. The listed cell IDs correspond to the IDs given in the original study that generated these data [3].*

References

[1] McInnes L, Healy J, Melville J. Umap: Uniform manifold approximation and projection for dimension reduction. arXiv preprint arXiv:180203426. 2018.

[2] Bartlett TE, Chandna S, Roy S. Stochastic networks theory to model single-cell genomic count data. arXiv preprint arXiv:230302498. 2023.

[3] Petropoulos S, Edsgärd D, Reinius B, Deng Q, Panula SP, Codeluppi S, et al. Single-cell RNA-seq reveals lineage and X chromosome dynamics in human preimplantation embryos. Cell. 2016;165(4):1012-26.

[4] Pal B, Chen Y, Vaillant F, Capaldo BD, Joyce R, Song X, et al. A single-cell RNA expression atlas of normal, preneoplastic and tumorigenic states in the human breast. The EMBO journal. 2021;40(11):e107333.

[5] Nee K, Ma D, Nguyen QH, Pein M, Pervolarakis N, Insua-Rodríguez J, et al. Preneoplastic stromal cells promote BRCA1-mediated breast tumorigenesis. Nature genetics. 2023;55(4):595-606.

[6] Kumar T, Nee K, Wei R, He S, Nguyen QH, Bai S, et al. A spatially resolved single-cell genomic atlas of the adult human breast. Nature. 2023;620(7972):181-91.

[7] Murrow LM, Weber RJ, Caruso JA, McGinnis CS, Phong K, Gascard P, et al. Mapping hormone-regulated cell-cell interaction networks in the human breast at single-cell resolution. Cell systems. 2022;13(8):644-64.

[8] Gray GK, Li CMC, Rosenbluth JM, Selfors LM, Girnius N, Lin JR, et al. A human breast atlas integrating single-cell proteomics and transcriptomics. Developmental cell. 2022;57(11):1400-20.

[9] Reed AD, Pensa S, Steif A, Stenning J, Kunz DJ, Porter LJ, et al. A single-cell atlas enables mapping of homeostatic cellular shifts in the adult human breast. Nature genetics. 2024;56(4):652-62.

[10] Twigger AJ, Engelbrecht LK, Bach K, Schultz-Pernice I, Pensa S, Stenning J, et al. Transcriptional changes in the mammary gland during lactation revealed by single cell sequencing of cells from human milk. Nature communications. 2022;13(1):562.

[11] Li Z, Schulz MH, Look T, Begemann M, Zenke M, Costa IG. Identification of transcription factor binding sites using ATAC-seq. Genome biology. 2019;20(1):1-21.

[12] Network CGA, et al. Comprehensive molecular portraits of human breast tumours. Nature. 2012;490(7418):61-70.
